# Evaluation of underreporting of salmonellosis and shigellosis hospitalised cases in Greece, 2011: results of a capture-recapture study and a hospital registry review

**DOI:** 10.1186/1471-2458-13-875

**Published:** 2013-09-23

**Authors:** Kassiani Mellou, Theologia Sideroglou, Athina Kallimani, Maria Potamiti-Komi, Danai Pervanidou, Eleni Lillakou, Theano Georgakopoulou, Georgia Mandilara, Maria Lambiri, Alkiviadis Vatopoulos, Christos Hadjichristodoulou

**Affiliations:** 1 Department of Hygiene and Epidemiology, University of Thessaly; 2 Hellenic Center for Disease Control and Prevention (HCDCP), 3-5 Agrafon Str, Marousi, Athens, Greece; 3 National Reference Laboratory for Salmonella and Shigella, Central Public Health Laboratory, National School of Public Health & Hellenic Center for Disease Control and Prevention (HCDCP), Vari, Greece

**Keywords:** Completeness, Evaluation, Underreporting, Surveillance, Capture recapture, *Salmonella*, *Shigella*

## Abstract

**Background:**

Salmonellosis and shigellosis are mandatorily notifiable diseases in Greece. Underreporting of both diseases has been postulated but there has not been any national study to quantify it. The objective of this study was to: a) estimate underreporting of hospitalised cases at public Greek hospitals in 2011 with a capture-recapture (C-RC) study, b) evaluate the accuracy of this estimation, c) investigate the possible impact of specific factors on notification rates, and d) estimate community incidence of both diseases.

**Methods:**

The mandatory notification system database and the database of the National Reference Laboratory for *Salmonella* and *Shigella* (NRLSS) were used in the C-RC study. The estimated total number of cases was compared with the actual number found by using the hospital records of the microbiological laboratories. Underreporting was also estimated by patients’ age-group, sex, type of hospital, region and month of notification. Assessment of the community incidence was based on the extrapolation of the hospitalisation rate of the diseases in Europe.

**Results:**

The estimated underreporting of salmonellosis and shigellosis cases through the C-RC study was 47.7% and 52.0%, respectively. The reporting rate of salmonellosis significantly varied between the thirteen regions of the country from 8.3% to 95.6% (median: 28.4%). Age and sex were not related to the probability of reporting. The notification rate did not significantly differ between urban and rural areas, however, large university hospitals had a higher underreporting rate than district hospitals (p-value < 0.001). The actual underreporting, based on the hospital records review, was close to the estimated via the C-RC study; 52.8% for salmonellosis and 58.4% for shigellosis. The predicted community incidence of salmonellosis ranged from 312 to 936 and of shigellosis from 35 to 104 cases per 100,000 population.

**Conclusions:**

Underreporting was higher than that reported by other countries and factors associated with underreporting should be further explored. C-RC analysis seems to be a useful tool for the assessment of the underreporting of hospitalised cases. National data on underreporting and under-ascertainment rate are needed for assessing the accuracy of the estimation of the community burden of the diseases.

## Background

Salmonellosis and shigellosis are included in the list of the 45 mandatory notifiable diseases in Greece. Salmonellosis is one of the most frequently reported diseases of the Mandatory Notification System (MNS) even though the notification rate has gradually decreased from 12 cases per 100,000 population in 2004 to 3.6 cases per 100,000 population in 2012 [1]. The notification rate of shigellosis has been quite stable during the same period (8 cases per 1,000,000 population in 2012) [1].

Based on- the European Centre for Disease Prevention and Control data (ECDC), the annual reported incidence for both diseases substantially differs among European countries [2]. This difference can only partially be attributed to differences in morbidity among countries and reflects, to some extent, the different ascertainment and reporting rates of the diseases [3].

Apart from few, restricted to small geographical areas of the country, studies [4], no nation-wide study has been conducted in Greece to estimate completeness of reporting or external completeness of MNS for salmonellosis and shigellosis in an objective and measurable way.

The best method to estimate surveillance systems’ completeness is to use an external data source as a reference and compare the number of notified cases to the number of cases included in the reference data source [5]. However, such a data source that can be used as a “gold standard” is not always available or accessible and alternative methods, such as capture-recapture (C-RC) analysis, have been proposed for estimating underreporting of surveillance systems [6-8].

The core idea of C-RC studies is to sample and identify cases of a disease in a population and then resample the population to see what fraction of cases in the second sample were, also, identified in the first one [9]. In this way, the fraction of cases not found in neither sample, as well as the level of underreporting of each data source (or combinations of them) can be calculated. The C-RC method, that was initially developed to estimate the size of wildlife populations [10], has been used for estimating underreporting of chronic diseases, as well as for tuberculosis, meningococcal disease, and other infectious diseases [11-14].

The objective of this study was to: a) estimate underreporting of hospitalised salmonellosis and shigellosis cases from public Greek hospitals to the MNS in 2011 via a C-RC study, b) evaluate the accuracy of this estimation, c) identify factors related to low notification rates in order to implement the appropriate correction measures for the improvement of the system, and d) assess the community incidence of both diseases.

## Methods

### Capture-recapture study

**Figure 1:**
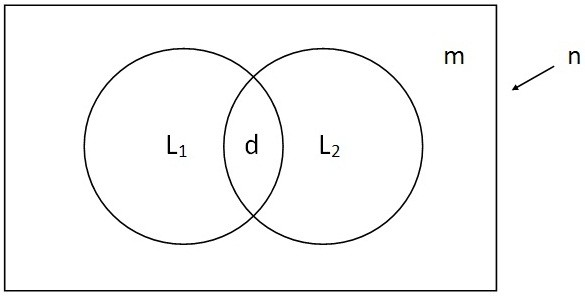
**Schematic description of the distribution of the total number of cases in a two-source capture-recapture model (Venn diagram).** n is the total number of cases in the population, L1 and L2 the number of cases captured by each one of the two systems, m the number of missing cases from both systems and d the duplicates.

In Greece, it is mandatory for all physicians, in public and private sector, to report salmonellosis and shigellosis cases to the local public health authorities and to the Hellenic Center for Disease Control and Prevention (HCDCP) via fax. Data entry takes place at HCDCP where cases are classified according to the 2008 EU definitions (Commission Decision 2008/426/EC). The notification form contains the name and demographic characteristics of cases (sex, date of birth, place of residence), clinical symptoms, laboratory data and the possible epidemiological link with other cases. In parallel, the serotypes of salmonellosis and species of shigellosis are monitored via the National Reference Laboratory for *Salmonella* and *Shigella* (NRLSS) surveillance system. Microbiological laboratories send isolates to NRLSS accompanied with a short form that includes the name and demographics (sex, date of birth, region) of the patient, and the date of specimen collection. Public hospitals are requested to send all isolates to NRLSS. In practice, many of the cases that are reported to MNS are not included in the NRLSS dataset and vice versa. The schematic description of the distribution of the total number of cases in a two-source C-RC model (Venn diagram) is presented in Figure 1.

The conducted C-RC study included all public hospitals of the country. Private hospitals were excluded since the number of reported salmonellosis and shigellosis cases by private hospitals in 2011 was very small (36 and 0 cases, respectively). Only hospitalised salmonellosis (typhoid and non-typhoid) and shigellosis cases were included in the analysis. Probable cases that had been reported to MNS without laboratory confirmation were excluded.

The datasets from the two systems were retrieved and data were checked for duplicates. Cases were matched using the variables “name” and “date of birth”. In those cases with inconclusive/unclear matching, the hospital was used as a third matching criterion. Matching was a continuous process throughout 2011. Data from the NRLSS were sent to the HCDCP every two weeks and matching was performed in order to incorporate the laboratory system information to the MNS database. In case there were two or more isolates from the same patient in the database of NRLSS, only the first one was included for the purposes of this study. Isolates sent to NRLSS from private sector were excluded.

No special permission for using the data was needed. HCDCP is the competent authority for surveillance of communicable diseases according to Greek legislation and has been officially authorised to receive, treat and temporarily store personal data of infectious diseases cases by the Greek Authority for Personal Data Protection. This study was conducted in the context of the evaluation of the surveillance system by the Department of Epidemiological Surveillance and Intervention of HCDCP.

Personal data were used only for the purposes of the evaluation during the matching process. All the necessary measures to protect the confidentiality of personal data were taken during the whole process of evaluation (access to the data restricted to the personnel involved in data analysis, removal of personal data from the datasets after matching etc).

The total number of salmonellosis and shigellosis cases (n) was estimated with Chapman’s formula developed for two-list C-RC studies [15]:

$$n=\frac{\left(L1+1\right)\;\left(L2+1\right)}{d+1}-1$$

$$95\%\;\mathrm{CI}=n\pm 1.96\sqrt{\frac{\left(L1+1\right)\;\left(L2+1\right)\;\left(L1-d\right)\;\left(L2-d\right)}{{\left(d+1\right)}^{2}\left(d+2\right)}}$$

L1 is the number of cases in the NRLSS dataset, L2 is the number of cases reported to MNS, and d is the number of cases captured by both systems. Underreporting to MNS was then estimated. Underreporting rates were also estimated by geographical region of the country, month of notification, type of hospital (university/district), serotype/species patients’ age group (< 5, 5–14, 15–24, 25+ years) and sex.

### Hospital registry review

Review of the hospital registries and comparison of data to the results of the C-RC study was conducted because: a) a third data source for adjusting calculations was not available [16,17], b) researchers did not know what kind of estimations to anticipate since this was the first time such a study was conducted, and c) only few published C-RC studies had been conducted on foodborne diseases [18-20]. During the first semester of 2012, the microbiological laboratories of the public hospitals were asked to review their registries and count the number of positive cultures for *Salmonella* spp. and *Shigella* spp. in 2011. An official letter was sent to each hospital explaining the rational and the objectives of this review. Only aggregated data (number of positive stool cultures for *Salmonella* spp. and *Shigella* spp.) were collected and access to the hospitals’ databases was not needed. The number of positive cultures was actually the total number of cases diagnosed and hospitalised during that period. Two or more positive cultures from the same patient were considered as only one case. The difference between the number of positive cultures and the number of reported cases to MNS represented the missed cases of MNS. The calculated underreporting rates through hospital registry review were then compared with the estimated rates from the C-RC study.

### Assessment of the community incidence of salmonellosis and shigellosis

We assessed/predicted the community incidence of the diseases in Greece using the percentage of the hospitalised cases out of the total number of diagnosed salmonellosis and shigellosis cases (1–3%), based on the results of a recent European study [21].

## Results

### Capture-recapture study

Ninety-eight public hospitals that had the capacity to perform stool cultures were included in the C-RC study. Nineteen isolates sent to the reference laboratory by private laboratories and 86 sent by private hospitals were excluded.

In total, 481 (*L*_1_) *Salmonella* isolates were sent to NRLSS and 478 (*L*_2_) salmonellosis cases were reported to MNS. The number of matched cases was 251 (d). The estimated total number of hospitalised salmonellosis cases in 2011 was 915 (95% CI: 861-969). Similarly, the estimated number for shigellosis was 98 (95% CI: 67-129), since 28 isolates were included in the NRLSS dataset (*L*_1_), 47 cases were reported (*L*_2_), and 13 cases were matched (d). The estimated underreporting of salmonellosis and shigellosis cases was 47.7% and 52.0%, respectively.

The underreporting rate of salmonellosis to MNS significantly varied between the thirteen regions of the country and ranged from 8.3% to 95.6% (median: 28.4%). Large university hospitals had significantly higher underreporting rate than district hospitals (p-value < 0.001). No specific pattern was identified in the monthly fluctuation of underreporting. Patients’ age and sex were not associated with the notification rate of the diseases.

### Hospital registry review

Based on the registry review of the 98 hospitals, the actual number of hospitalised salmonellosis and shigellosis cases (number of positive stool samples) was 1012 and 113, respectively, leading to an underreporting of 52.8% for salmonellosis and 58.4% for shigellosis (Table 1).

**Table 1:** Underreporting of salmonellosis and shigellosis hospitalised cases, Greece, 2011

	***Salmonellosis***	***Shigellosis***
**Notification**		
Number of cases–MNS*	478	47
Number of isolates–NRLSS**	481	28
**Capture-recapture study**		
Estimated total number of cases (95% CI†)	915 (861–969)	98 (67–129)
Estimated underreporting–MNS (95% CI†)	47.7% (41.8–53.6%)	52.0% (29.8–63.5)
**Hospital registry review**		
Number of diagnosed cases (positive cultures)	1012	113
Number of cases not reported to MNS	534 (1012–478)	66 (113–47)
Underreporting-MNS	534/1012 (52.8%)	66/113 (58.4%)

Results of a capture-recapture study and a hospital registry review.

*MNS: Mandatory Notification System.

**NRLSS: National Reference Laboratory for *Salmonella* spp. and *Shigella* spp*.*

† CI: confidence interval.

### Assessment of the community incidence of salmonellosis and shigellosis

The predicted community incidence of salmonellosis for 2011 ranged from 312 to 936 cases per 100,000 population, and the incidence of shigellosis ranged from 35 to 104 cases per 100,000 population.

## Discussion

The main purpose of public health surveillance is to provide timely, scientifically sound evidence to stakeholders and decision makers, in order to make decisions for improving public health in their jurisdiction [22]. Completeness of reporting is an important attribute to achieve this objective. The surveillance system should be appropriately inclusive and the reported cases should also represent the complete list of eligible persons. In practice, it is a very challenging task to capture all cases of a disease in the population; thus, evaluation of completeness is required on a regular basis [22]. Quantification of underreporting allows the establishment of a baseline for data quality and the identification of areas for improvement [5,23]. It, also, allows comparisons between countries, since diversity of the health care and surveillance systems handicaps data comparability.

The estimated reporting rates of salmonellosis and shigellosis in this study were similar to previous estimations in the country at a local level [4] and to the estimations of reporting rates in Spain [24]. Other European countries have much higher completeness that has been reported to reach almost 100% when an electronic reporting system is in place; 95.8% in Sweden [25], 99.0% in Germany [21] and 82.0% in Italy [21].

The estimations of the C-RC study were similar to the results of the hospital registry review. Authors believe that the three of the four assumptions of C-RC studies were not seriously violated; the population was a closed, well-defined “cohort”, cases of the surveillance systems were identified and matched, and all cases had the same probability to be reported to each of the systems (equal catchability) [16,17]. On the other hand, there might have been positive dependence between the data leading to an underestimation of underreporting. In better staffed hospitals, for example, cases that were reported to MNS might have been more likely to also be found in the NRLSS dataset [16,17]. However, positive dependence in this case was quite small as indicated by the comparison with the results of the registry review.

The results of this study and of similar studies can be used for better understanding the causes of underreporting of the diseases to MNS; for example, reporting from larger university hospitals may be impaired by the increased work load of personnel or by the lack of supervision and training on reporting processes. Geographical and seasonal differences, also, need further study. Due to the small number of reported cases, these parameters could not be assessed for shigellosis. Low internal completeness of the variable “serotype” for *Salmonella* and the variable “species” for *Shigella* of the MNS dataset did not allow the estimation of underreporting by serotype/species. The factors that mainly affect the completeness of surveillance systems and should be further studied are related to the health care system (lack of personnel, of technical support, etc.), the data providers (lack of interest or training, etc.) or the surveillance system (long or complicated reporting forms, lack of electronic reporting systems, etc.) [26,27]. Results of this study were disseminated to the hospitals accompanied with a request to all clinical doctors to systematically notify salmonellosis and shigellosis cases and to report possible problems of the reporting process.

Estimation of the diseases’ community incidence using the hospitalisation rate of the diseases reported by other European countries may not have been totally accurate; however this approach was decided since a nation-wide study on under-ascertainment of the two diseases is not available. Published estimations of the foodborne diseases’ community incidence in Greece are based on travelers [28,29] or data from other countries [30-32] and are subject to several biases. In addition, the available estimations refer to past data and may be outdated and no longer applicable [30]. Thus, we believe that the extrapolation of the hospitalisation rate based on recent data from other European countries with quite similar morbidity patterns with Greece -though not the optimum method- leads to a more accurate estimation than the already available.

Another limitation was that private hospitals were not included in the estimation. Based on the literature, admissions to private hospitals in Greece account for 16% of total admissions, but a specific estimation for salmonellosis and shigellosis cases is not available [33]. The range of the estimated community incidence of salmonellosis, though wide, was compatible to estimations from other European countries [34], higher than in Canada [26], Australia [31], and Netherlands [34] and lower than Poland [21].

## Conclusions

The conducted C-RC study led to a quite accurate estimation of the notification rates of salmonellosis and shigellosis without the need to collect new data. Consequently, this is deemed a tool that can be used for regular evaluation of MNS and for assessing the effectiveness of correction measures taken for the improvement of the system. However, the restrictions of C-RC studies should be kept in mind given the fact that a third source for adjusting calculations is not available [35].

Differences in the notification rate by geographical region and hospital can guide interventions for the improvement of notification.

Finally, for accurately estimating the community burden of salmonellosis and shigellosis, a nation-wide study on current underreporting and under-ascertainment rate of the diseases is needed.

## Competing interest

The authors declare that they have no competing interest.

## Authors’ contributions

All authors (KM, TS, AK, MPK, DP, EL, TG, GM, ML, AV, CH) have participated in the conception and design of the above evaluation, analysis and interpretation of the presented data, drafting and revision of the manuscript. They have all read and approved the final manuscript.

## Funding statement

This research received no specific grant from any funding agency in the public, commercial or not-for-profit sectors.

## Pre-publication history

The pre-publication history for this paper can be accessed here:

http://www.biomedcentral.com/1471-2458/13/875/prepub
